# Dermatofibrosarcoma Protuberans: Update on the Diagnosis and Treatment

**DOI:** 10.3390/jcm9061752

**Published:** 2020-06-05

**Authors:** Xingpei Hao, Steven D. Billings, Fangbai Wu, Todd W. Stultz, Gary W. Procop, Gene Mirkin, Allison T. Vidimos

**Affiliations:** 1 Foot and Ankle Specialists of the Mid-Atlantic, Rockville, MD 20850, USA; 2 Department of Pathology, Cleveland Clinic, Cleveland, OH 44195, USA; billins@ccf.org (S.D.B.); procopg@ccf.org (G.W.P.); 3 Department of Radiology, Cleveland Clinic, Cleveland, OH 44195, USA; WUF@ccf.org; 4 Imaging Institute, Section of Neuroradiology, Cleveland Clinic, Cleveland, OH 44195, USA; stultzt@ccf.org; 5 Department of Dermatology, Cleveland Clinic, Cleveland, OH 44195, USA

**Keywords:** dermatofibrosarcoma protuberans, wide local excision, Mohs micrographic surgery, targeted therapy, pathology, tumorigenesis

## Abstract

Dermatofibrosarcoma protuberans (DFSP) is a slow growing, low- to intermediate-grade dermal soft-tissue tumor. It has a high local recurrence rate but low metastatic potential. It is characterized by a uniform spindle cell arrangement, classically with a storiform pattern and CD34 immunoreactivity. The histomorphology and immunophenotype overlap with a broad range of other neoplasms. The standard treatment is complete surgical excision. The surgical procedures include wide local excision (WLE) with tumor free margins, Mohs micrographic surgery (MMS) and amputation. Unresectable DFSPs are treated with radiation therapy and/or targeted therapy. DFSP has characteristic t(17; 22) (q22; q13), resulting in a *COL1A1- PDGFB* fusion transcripts in more than 90% of DFSPs. Molecular detection of the gene rearrangement or fusion transcripts is helpful for the diagnosis of patients with atypical morphology and for screening candidates for targeted therapy with tyrosine kinase inhibitors. The aims of the present review are to update the clinical presentation, tumorigenesis and histopathology of DFSP and its variants for diagnosis and differential diagnosis from other benign and malignant tumors, to compare the advantages and drawbacks of WLE and MMS, to propose the baseline for selecting surgical procedure based on tumor’s location, size, stage and relationship with surrounding soft tissue and bone structures, and to provide a biologic rationale for the systemic therapy. We further propose a modified clinical staging system of DFSP and a surveillance program for the patients after surgical excision.

## 1. Introduction

Dermatofibrosarcoma protuberans (DFSP), derived from dermal fibroblasts, was initially characterized as keloid sarcoma. It was named as DFSP by Hoffman in 1925 [1]. Clinically, it is a slow-growing, low- to intermediate-grade malignant sarcoma and frequently occurs in middle-aged adults. Histopathologically, it is composed of uniform spindle cell fascicles growing in a storiform pattern with multiple variants and with strong and diffuse CD34 immunoreactivity. However, its spindle cell morphology and CD34 immunostaining pattern are overlapped with other benign and malignant lesions, which must be differentiated from. Ultrastructurally, DFSP is characterized by stellate or spindle cells, with long, slender, ramified cell processes joined by primitive junctions, which are similar to dermal dendrocytes [2]. Cytogenetically, more than 90% of DFSP have a t(17; 22) (q22; q13), leading to the formation of *COL1A1-PDGFB* fusion transcripts. Molecular detection of gene rearrangements or fusion transcripts is beneficial not only for the diagnosis in cases without typical morphology, but also for screening patients who are candidates for using imatinib mesylate (a tyrosine kinase inhibitor that affects PDGFβR). This provides neoadjuvant targeted therapy for the patients with unresectable, recurrent or metastatic DFSP [3]. The standard treatment of resectable DFSPs is complete surgical excision with either wide local excision with tumor free margins or Mohs micrographic surgery, or, rarely, amputation. Each procedure has advantages and drawbacks. Unresectable DFSPs are treated with radiation therapy and/or targeted therapy. Accordingly, we update the DFSP’s clinical manifestations, histological features with its variants, diagnosis, differential diagnosis and tumorigenesis. We discuss the advantages and drawbacks of different surgical procedures for resectable tumors and the width of margin resection. We review the biologic rationales of radiation therapy and/or targeted therapy for unresectable tumors. We also propose a modified staging system of DFSP for clinical practice and a surveillance program to monitor local recurrence and metastasis after surgical excision.

## 2. Epidemiology

DFSP is an uncommon, indolent dermal soft-tissue sarcoma that accounts for less than 0.1% of all malignancies and less than 1% of all soft-tissue sarcomas [4,5,6]. Two large epidemiological studies in the United States demonstrated that the annual incidence of DFSP was 4.2 per million people studied in a 30-year period from 1973 to 2002 [7], and 4.1 per million people in a 10-year period between 2000 and 2010 [8]. The incidence of DFSP is higher in women than men, and higher in African-American than white patients [4,5]. It most frequently occurs in young and middle-aged patients, between 25 and 45 years of age, with a mean age between 40 to 43 years [9,10]. However, patient’s age ranged widely from infancy to the elderly [11,12,13].

## 3. Pathogenesis

Cytogenetic and molecular studies have demonstrated that more than 90% of DFSPs are characterized by either supernumerary ring chromosomes derived from chromosomes 17 and 22 or chromosomal translocation t(17; 22) (q22; q13), resulting in the fusion of collagen type 1-alpha 1(*COL1A1* at 17q22) and platelet-derived growth factor beta (*PDGFB* at 22q13) genes. The gene fusion places the *PDGFB* gene under the control of the *COL1A1* promoter [14,15,16], leading to PDGFβ overexpression and dimerization, and subsequently resulting in continuous activation of the PDGF receptor β protein-tyrosine kinase [17]. Interaction of PDGFβ and PDGF receptor β is involved in multiple signaling pathways including Ras mitogen-activated protein kinases (RAS-MARK) and phosphatidylinositol 3-kinase-akt-rapamycin (mTOR) (PI3K-AKT-mTOR) [18,19,20,21]. Correspondingly, increased expression of the phosphorylated Akt-mTOR pathway proteins including Akt, mTOR, 4EBP1, and S6RP and phosphor-PDGFRα/β have been demonstrated in about half of DFSP tissues by immunoperoxidase studies [21], suggesting that Akt-mTOR pathways are involved in the tumorigenesis of DFSP. Gene fusion transcript of *COL1A1*−*PDGFB* can be detected by either fluorescent in situ hybridization (FISH) or multiplex reverse transcriptase-polymerase chain reaction (RT-PCR) in formalin fixed, paraffin embedded tissues [22,23]. These are helpful for the diagnosis, differential diagnosis and guiding treatment of DFSP [24], especially when the tumor’s histomorphology is not typical or when the tyrosine kinase inhibitors are considered for treatment. The *COL1A1-PDGFB* fusion transcript cannot be detected in about 8% of DFSPs [15]. FISH analysis revealed genetic translocations involving the *CSPG2* gene at 5q14.3 and *PTK2B* gene at 8p21.2 in a patient of DFSP without the *COL1A1-PDGFB* fusion transcript [16]. Other reported genetic translocations in DFSP include *COL1A2-PDGFB* [3], *COL6A3-PDGFD* [25] and elastin microfibril interface 2 (*EMILIN2*)-*PDGFD* [26]. Moreover, p53 mutation and overexpression, murine double minute 2 (MDM2) overexpression were reported in fibrosarcomatous variant of DFSP [17,27]. Furthermore, DFSPs were reported in patients with immunodeficiency disorders, including X-linked agammaglobulinemia [28], adenosine deaminase-deficient severe combined immune deficiency [29], ataxia telangiectasia syndrome [30] and HIV infection [31]. In addition, pregnancy may increase the risk for the development of DFSP [32]. These data suggest that multiple factors including oncogenes, tumor suppressor genes and immunodeficiency are involved in the development of DFSP. Further investigation is required to understand the relationship of these risk factors to the development of DFSP.

## 4. Clinical Characteristics

**Figure 1:**
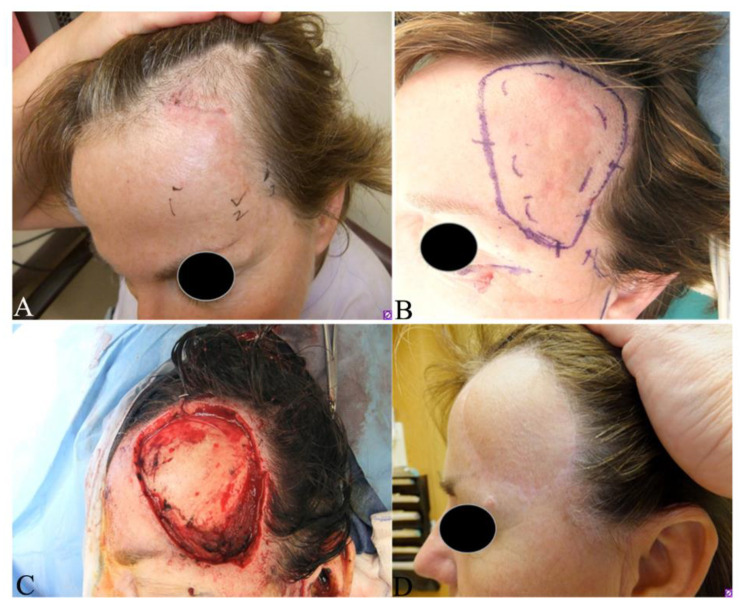
Clinical presentation and Mohs micrographic surgery (MMS) of a female patient with a primary fibrosarcomatous–dermatofibrosarcoma protuberans. (**A**) Asymptomatic, ill-defined plaque on her left frontal scalp, with scouting biopsies at inferior aspect; preoperative magnetic resonance imaging showed no bony invasion (Figure 3), PET CT showed no local or distant metastases; (**B**) surgical marks for MMS. The inner dashed lines indicate the palpable and pathologically positive tumor boundaries while the solid outer lines indicate the surgical incision with the first Mohs layer; (**C**) wound after first stage of Mohs surgery, all deep and peripheral margins were clear; (**D**) postoperative at 12 months following a free flap repair. Currently 9 years postop with no evidence of recurrence.

**Figure 2:**
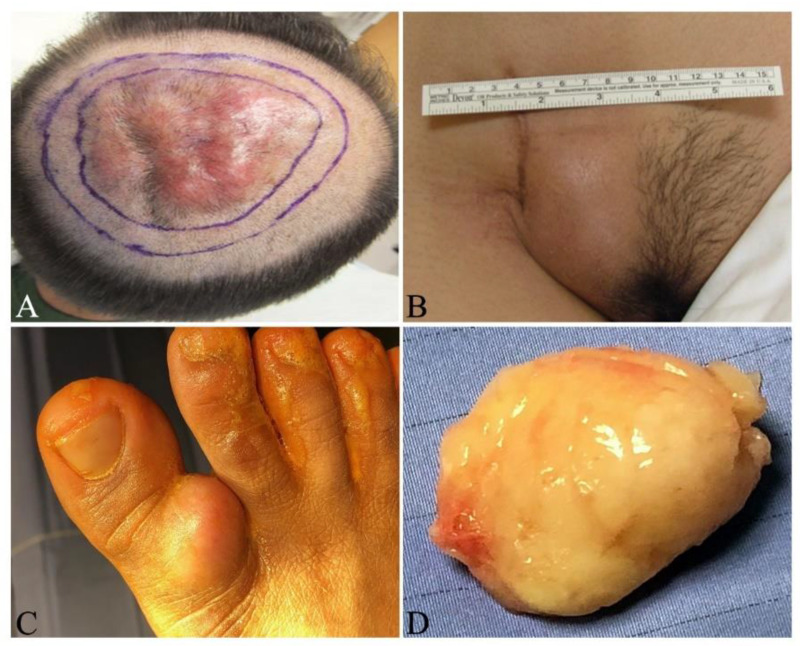
Clinical presentation of dermatofibrosarcoma protuberans. (**A**) Multiple, raised, erythematous, confluent nodules on the vertex of the scalp from an adult male. The inner circle indicates the palpable tumor boundary and the outer line indicates the surgical excision during Mohs surgery; **(B)** recurrent, skin colored, raised mass around the scar in the right lower abdomen from an adult female; (**C**) skin-colored, raised tumor on the lateral side of the right hallux from an adult male; (**D**) white to yellow, solid, fish flesh-like soft tissue mass enucleated from figure C with patient’s agreement. Tumor cells were observed on all margins. The patient was referred to an oncologist for radiation therapy since any further invasive surgery was declined.

In early stages of DFSP, the patient typically notices a slow-growing, small, firm, painless, skin-colored dermal plaque (Figure 1A,B), subcutaneous thickening or atrophic non-protuberant lesion [33,34]. Early pediatric DFSPs were classified into four variants: (1). Small palpable nodules slowly forming a confluent sclerotic plaque; (2). Keloid-like homogenous cutaneous thickening plaque; (3). Tumor ab initio and (4). Atrophic plaque [35]. Congenital DFSPs were reported as either erythematous atrophic plaque or irregular shaped, pigmented macula resembling melanocytic nevus after birth [34,36]. These congenital DFSPs carried the *COL1A1-PDGFB* fusion detected by either RT-PCR or FISH [34,36]. The early non-protuberant lesions gradually enlarge to form protuberant, indurated, reddish-blue or violaceous nodules in protuberant stages (Figure 2A–C). If untreated, the tumors can locally invade more deeply into the fascia, muscle, periosteum, bone and occasionally metastasize to other organs in advanced stages [37]. The tumor cells most frequently metastasize to lung, brain, bone, visceral organs, lymph nodes and soft tissues [13,38,39,40]. This insidious growing process takes several months or years to as long as 60 years [13,33,34]. The reported tumor sizes vary in a wide range from 0.5 to >10 cm in diameter, with a mean of 2–3.5 cm [41,42]. Most DFSPs are localized on the trunk (40–50%), followed by proximal extremities (30–40%), and then the head and neck (10–15%) [43,44,45]. Infrequently documented cases have been reported on the toes [22,46,47,48,49], scalp [50], breast [51,52,53] and vulva [54].

## 5. Clinical and Imaging Evaluation

In addition to a thorough history and physical examination, diagnosis of suspected DFSP ideally requires a generous biopsy (punch biopsy or excisional biopsy) for a pathologic diagnosis. The pathology report should note the presence of fibrosarcomatous change or other high-risk features. Examination of lymph nodes and imaging studies are important for staging and surgical planning.

**Figure 3:**
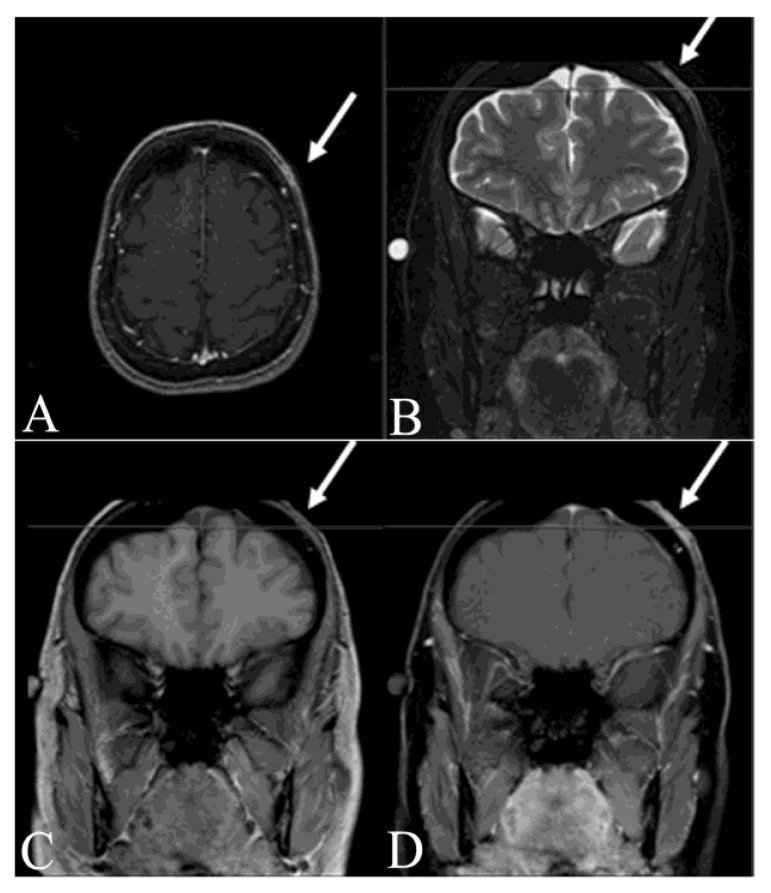
Magnetic resonance imaging of fibrosarcomatous-dermatofibrosarcoma protuberans on the left forehead from patient in Figure 1. (**A**) Left scalp enhancement (arrow) in axial post-gadolinium thin slice image; (**B**) left scalp hyperintensive lesion without bony invasion (arrow) on coronal fat saturated T2-weighted image; (**C**) left scalp isointense lesion (arrow) before gadolinium on T1-weighted image; (**D**) left scalp hyperintense lesion (arrow) after gadolinium on T1-weighted image.

**Figure 4:**
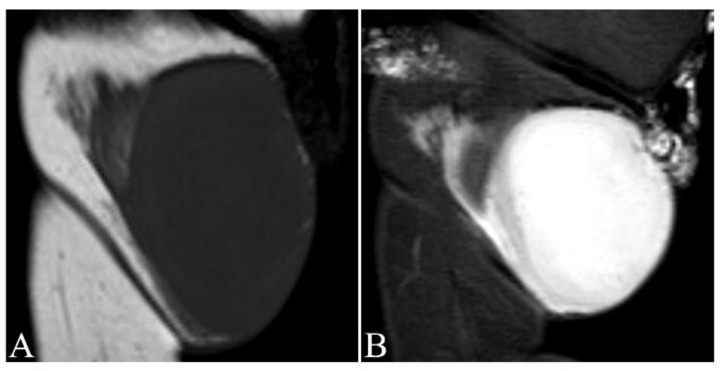
Magnetic resonance imaging (MRI) of dermatofibrosarcoma protuberans on the left thigh of an 8-month-old girl. (**A**) MR T1-weighted image exhibiting well-defined homogeneous isointense lesion; **(B)** T2-weighted image showing a well-defined mass with intermediate-to-marked homogeneous hyperintensity with infiltration of the adjacent fat plane and encasement of the gracilis muscle at the level of this image.

**Figure 5:**
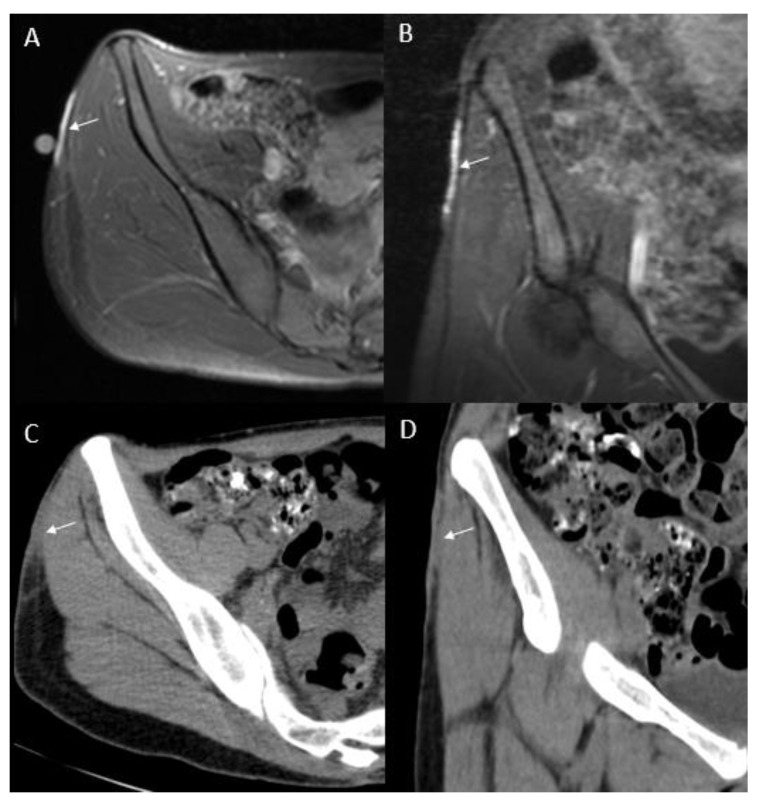
Magnetic resonance imaging (MRI) and computed tomography (CT) of a locally recurrent dermatofibrosarcoma protuberans (DFSP) of the right hip. (**A)** axial and (**B)** coronal MR T1-weighted fat suppressed post-contrast images of the right hip show enhancing tumor (arrows) diagnosed as a recurrent DFSP; (**C)** axial and (**D)** coronal corresponding images from non-contrast CT demonstrate soft tissue attenuation of the tumor (arrows).

Magnetic resonance imaging (MRI) delineates tumor’s size and extent, and its relationship with adjacent neuromuscular structures and bone (Figure 3, Figure 4 and Figure 5A,B). MRI is therefore recommended for pre-operative evaluation, surgical planning and follow-up for recurrence [55,56]. MR T1-weighted images exhibit well-defined homogeneous isointense lesions, while T2-weighted images show a well-defined subcutaneous soft tissue nodules or mass with intermediate-to-marked homogeneous hyperintensity to the surrounding muscular tissues (Figure 4A,B) [57]. Poorly defined irregular margins can be observed in some cases (Figure 4B) [58]. High frequency ultrasound can be used to evaluate the extent of the tumor involvement as well as provide biopsy guidance. The ultrasound of DFSP often appears as a hypoechoic superficial nodular mass [59,60]. Computed tomography (CT) reveals a solitary, subcutaneous lobular or nodular architecture and soft tissue attenuation and post-contrast enhancement (Figure 5C,D) [61]. Intratumoral non-enhancement areas in large tumors (>5 cm) may suggest necrosis and cystic degeneration. CT is useful to evaluate distant metastatic disease. The technique of 18F-fluorodeoxyglucose–positron emission tomography/computed tomography (^18^F-FDG PET/CT) has shown potential value in both identifying metastatic disease and evaluating treatment response [61,62,63]. X-rays have no role in the imaging of the primary DFSP.

## 6. Pathologic diagnosis

**Figure 6:**
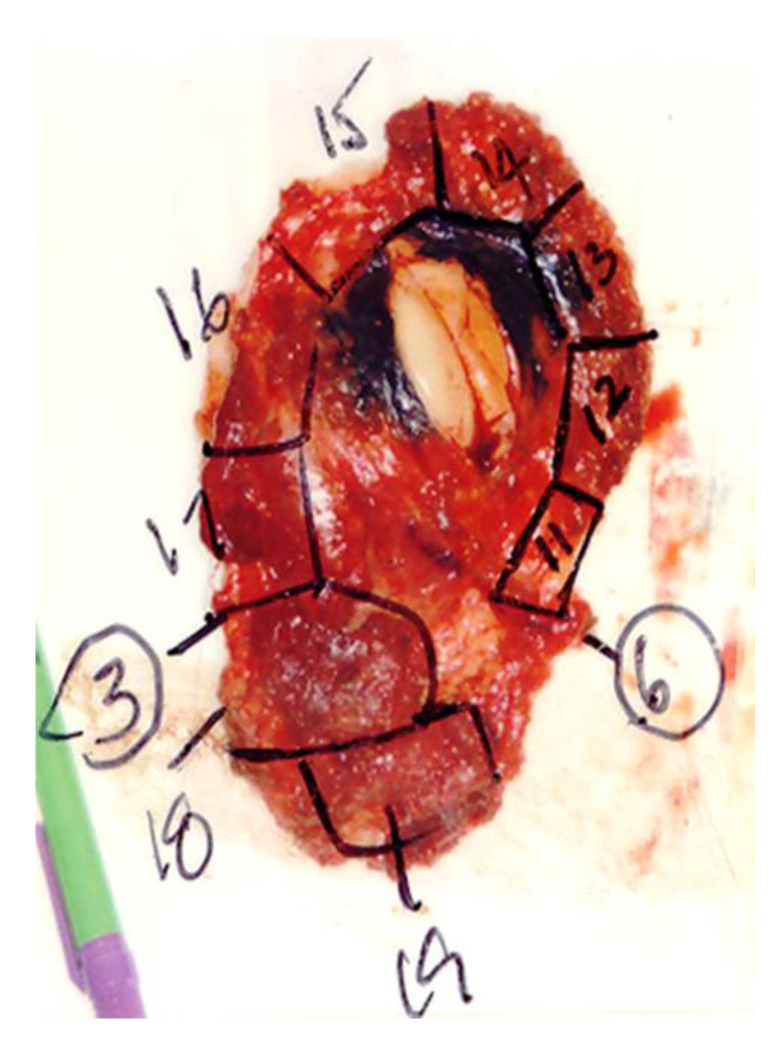
Photograph of pathologic grossing and mapping of the deep tumor margins during Mohs surgery for a dermatofibrosarcoma protuberans. All of the tumor margins were labeled and grossed to examine the residual tumor cells.

Grossly, DFSP is commonly a white to yellow color, poorly circumscribed, soft-tissue mass without a smooth outer surface. The cut surface is white to yellow, poorly encapsulated, solid and a fish flesh-like texture (Figure 2D and Figure 6). Hemorrhagic and/or cystic changes can be observed in larger tumors (>5 cm). All sides of margins need to be labeled and grossed to examine if any part of the margins contains tumor cells or not (Figure 6).

**Figure 7:**
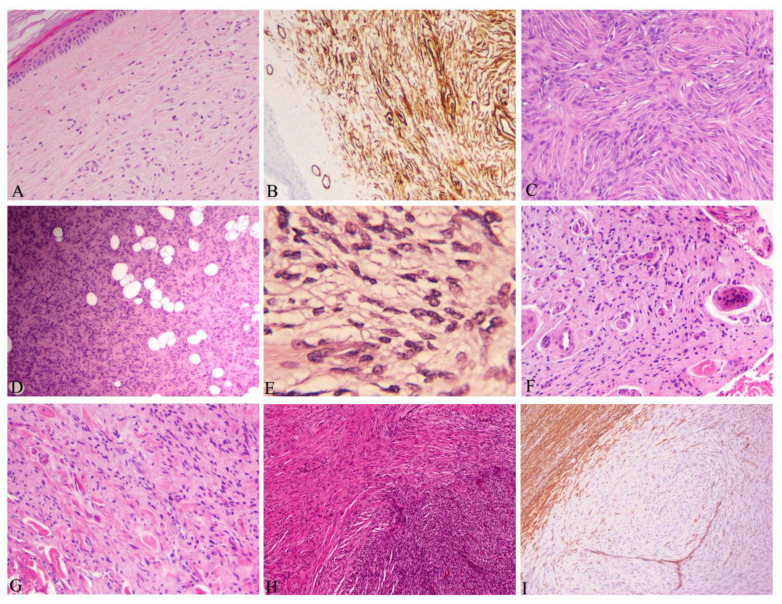
Histopathology of dermatofibrosarcoma protuberans. (**A**) Elongated spindle cells loosely scattered in the upper dermis without involving grenz zone in early stage (dermal plaque) (HE, 40 × 1); (**B**) diffuse and strong CD34 immunostaining in spindle cells (same case as A, DAB, 40 × 1); (**C**) dense spindle cells in a storiform (HE, 100 × 1); (**D**) spindle cells infiltrating into surrounding fatty tissues forming a honeycomb-like structure (HE, 40 × 1); (**E**) myxoid variant: Spindle cells in the myxoid stroma (HE, 100 × 1); (**F**) giant variant: Polymorphic and giant cells admixed with spindle cells (HE, 100 × 1); (**G**) sclerotic variant: Less than 50% of spindle cells in the hypocellular collagenous stroma (HE, 100 × 1); (**H**) fibrosarcomatous component (right lower part) with increased atypia of cellularity, hyperchromasia and mitosis in a transition from classic DFSP (upper left part) (HE, 100 × 1); (**I**) loss of CD34 expression in fibrosarcomatous components compared with classic DFSP part where CD34 was strongly expressed (upper left part, same case as H) (DAB, 100 × 1).

Histologically, DFSP is derived from fibroblasts in the dermis and subsequently it can infiltrate into the subcutaneous tissues or it can develop directly from subcutaneous tissues [64]. In non-protuberant stage I with dermal plaque or subcutaneous thickening, the elongated spindle cells are loosely scattered in the upper dermis without involving grenz zone (Figure 7A). In stage II and later with protuberant lesions, DFSP is typically featured with uniformly monomorphous spindle cells, with little atypia and mitotic activity, arranged in a storiform pattern, in the subcutaneous and dermal layers (Figure 7C). The cellular nuclei are elongated with mild hyperchromasia, small to inconspicuous nucleoli and low to moderate quantities of cytoplasm. The neoplastic cells often infiltrate into subcutaneous adipose tissue in a honeycomb pattern (Figure 7D). This poses challenge to determine the true extent of the tumor tissue. All margins need to be carefully grossed and examined for residual tumor cells (Figure 6). Immunohistochemically, spindle cells typically show strong and diffuse cytoplasmic expression of CD34 (Figure 7B), but negative expression for other immunohistochemical stains, such as alpha-smooth muscle actin, factor XIIIa, S-100 and melan-A. It should be noted that CD34 expression could be reduced or even lost in up to 45% of the fibrosarcomatous DFSP (Figure 7I) [13,65]. CD34 expression is not unique to DFSP. Other tumors, including spindle cell lipomas, fibromas, fibromyxomas and Kaposi sarcomas, also express CD34.

DFSPs have multiple histological variants including myxoid (Figure 7E), pigmented, giant cell (Figure 7F), giant cell fibroblastoma, granular cell, sclerotic (Figure 7G) and fibrosarcomatous (FS) component (Figure 7H, Table 1). These variants reflect the morphologic heterogeneity which is associated with the spindle cell differentiation during tumor development. They do not bear significant clinical manifestations and outcomes, except for the FS variant with increased risk of local recurrence and metastatic potential [28].

DFSP without FS component is categorized as classic or conventional DFSP, accounting for 80–90% of all DFSPs [66]. It is considered a low-grade malignancy with a propensity of local recurrences following resection, but almost no metastatic potential [67,68]. FS-DFSP is featured with atypical spindle cells with significantly increased mitotic activity arranged in a herringbone pattern (Figure 7H), often with reduced or even loss of CD34 expression (Figure 7I), but increased Ki-67 expression (a marker of cellular proliferation) [13]. FS-DFSPs consisting of 10–20% of DFSPs are considered intermediate-grade sarcoma with a higher metastatic risk (5–15%) [13,67]. Analysis of 24 reports containing 1422 patients with DFSP and 225 with FS-DFSP revealed that FS-DFSPs, compared with classic DFSPs, had a significantly higher risks of local recurrence (29.8% vs 13.7%, risk ratio 2.2 (95% confidence interval 1.7–2.9)); metastasis (14.4% vs 1.1%, risk ratio 5.5 (95% confidence interval 4.3–7.0)); and death from disease (14.7% vs 0.8%, risk ratio 6.2 (95% confidence interval 5.0–7.8)) [66].

## 7. Differential diagnosis

Similarities in clinical manifestations and overlaps in histopathologic and CD34 immunostaining profiles with other tumors require DFSP to be differentiated from other benign and malignant lesions including dermatofibroma, schwannoma, cutaneous neurofibroma, solitary fibrous tumor, intradermal spindle cell lipoma and spindle cell or desmoplastic melanoma. Detailed evaluation of the clinical presentation and morphologic features with immunohistochemistry are needed to make an accurate diagnosis (Table 2). Difficult cases can be further tested by molecular techniques including FISH and RT-PCR to detect gene rearrangements and gene fusion transcripts in formalin fixed, paraffin embedded tumor tissues [79].

## 8. Clinical Staging System

No standard staging system of DFSP is available [86]. We propose a modified staging system of DFSP based on European consensus-based interdisciplinary guideline [86], the progression of DFSPs’ tumorigenesis and clinical presentation, as shown in Table 3. This staging system is useful for treatment.

## 9. Treatment

### 9.1. Treatment of Resectable DFSP

Surgical excision is the standard treatment of DFSP including stage I and II, even III and IV whenever feasible. Wide undermining following surgical excision is not advisable as it may seed tumor in incomplete resections and also may cause difficulty in interpreting subsequent re-excisions. Initial resected tumors with positive margins or relapsed/recurrent tumors need to be further resected to achieve wide clear margins whenever possible [41]. Surgical reconstruction should be delayed until all margins are confirmed negative by complete peripheral and deep margin examination. If concern exists for positive surgical margins following wide excision, a split thickness skin graft may be placed to facilitate monitoring for recurrence.

**Figure 9:**
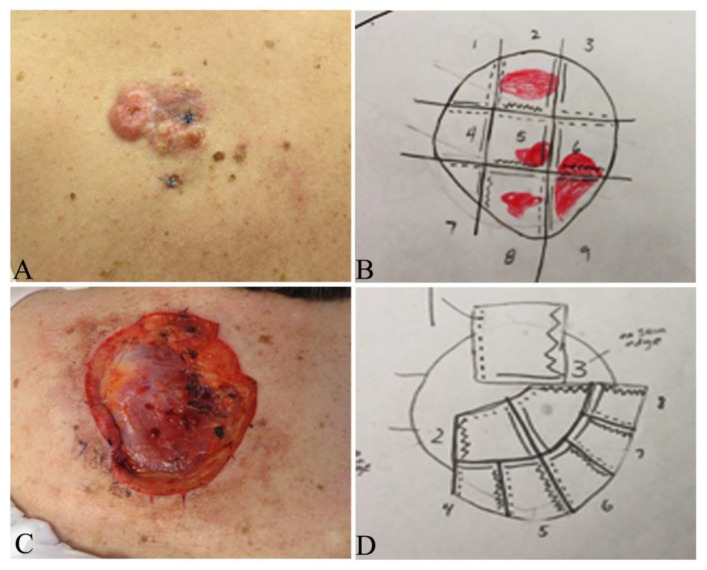
Clinical presentation and Mohs map in a patient with a primary dermatofibrosarcoma protuberans on the back. (**A**) Several irregular, firm confluent nodules on the left upper back; (**B**) Mohs map of A for the first stage dissection; (**C**) surgical surface after the primary tumor was dissected out; (**D**) Mohs map of the second stage dissection.

Surgical techniques include wide local excision (WLE) with tumor-free margins, Mohs micrographic surgery (MMS) (Figure 1B–D and Figure 9B–D), partial or total amputation if the tumor is located on the upper or lower digits [28,87,88]. Both WLE and MMS are used in the clinical practice and each has advantages and drawbacks, as summarized in Table 4.

Multiple studies have shown that MMS can significantly lower the risk of recurrence of DFSP, compared with WLE [88,90,91]. A comprehensive retrospective meta-analysis involving 684 patients of DFSP published on Medline from 2008 to 2018 revealed that the recurrence rates of DFSP treated with WLE and MMS were 9.10% and 2.72%, respectively, with mean follow-ups of 5.32 years for both groups [92]. Lowe et al. reported the Mayo Clinic experience illustrating a 30.8% recurrence rate following WLE and 3.0% with Mohs surgery; primary closure was performed following MMS in 73% of cases, vs. flaps, graft and other closures in 52% of the WLE cases [90]. Multidisciplinary management is advantageous with infiltrative DFSP of the head and neck as well as large tumors on the trunk, where the Mohs surgeon does the tumor mapping and histologic examination of all tumor margins in concert with another ablative surgeon (Figure 9) [93].

The width of the tumor free margins is an important factor to be considered for complete excision for both WLE and MMS. However, no agreement on optimal width of margins is available. NCCN guidelines (version 1.2020) suggest 2–4 cm lateral margins from the tumor and the excision of the investing fascia to remove any infiltrating tumor in WLE [94]. Ratner et al analyzed records of 58 patients with primary and recurrent DFSP treated with Mohs surgery and reported that 70% had positive margins with a 1 cm margin, 39.7% with 2 cm, 15.5% with 3 cm and 5.2% with 5 cm margins [95]. The reported local recurrence (LR) rates with width margins varied widely. Monnier et al. reported a LR of 47% in 66 patients with width margins less than 3 cm at a mean follow-up of 32 months [96], whereas Farma et al. reported LR of 0.9% in 206 patients using 2 cm margins at a follow-up of 64 months [97]. Snow at al recently reported a LR of 1% and a distal recurrence of 1% in 98 patients at a follow-up of 53 months [98]. Among them, 44 patients with microscopically incompletely excised DFSP were treated with conservative re-excision with a mean width of 1.54 cm, and 54 patients with primary tumors were excised with a mean margin of 2.4 cm [98]. Harati analyzed 68 patients with DFSP and observed that 2 cm width margins of normal tissues in primary tumors and in incompletely resected tissues around the scar yielded median negative margins of 0.35 cm and 0.8 cm [99], respectively. These data suggest that narrower margins may be good enough to prevent local recurrence. Mullen suggests to choose WLE with a 1.0 to 1.5 cm safe margins from tumor boundary for most DFSPs on the trunk or extremities since the tumor can be excised in a single stage to achieve excellent cosmetic and functional outcomes, whereas MMS should be selected for relatively small DFSPs in cosmetically sensitive regions including face, scalp (Figure 1B–D) and neck, for best tissue preservation, cosmetic and functional outcomes [89].

Dissection of the tumor bed should be based on the infiltrating depth of the tumor. Deep tumors (Stage IIB) should be excised to include the underlying investing fascia of muscle or pericranium whereas superficial tumors (Stage I + IIA) may be directly excised without dissecting underlying fascia. Since WLE usually requires wide and deep excision from the periphery of the tumor, this makes it difficult to treat the DFSP on digits due to the limited space and the complex structures surrounding the toes and fingers. Mohs surgery may allow tissue sparing for DFSP of the digits, however if tumor extends to periosteum, partial or total amputation of the involved digit will be necessary to obtain tumor-free margins and allows for primary closure with faster return to function [22]. When the patient does not agree with amputation due to concern of functional and cosmetic impairments, tumor can be directly enucleated as demonstrated in Figure 2C,D. The patient needs to be referred to oncologists for further radiation therapy and/or targeted therapy. In surgical practice, selection of which procedure for individual patient must be based on tumor location, size, stage, relationship with surrounding neuro-muscular and bone structures, cosmetic and functional requirements, cost to the patient and the medical resources.

### 9.2. Treatment of FS-DFSP

The FS-DFSP variant is a much more aggressive tumor with local recurrence in more than 50% of patients and metastasis in 10% to 15% of patients [13,100]. FS-DFSP treatment needs consultation with a multidisciplinary specialized soft-tissue sarcoma tumor board [86]. Aggressive treatments for FS-DFSP include adequate WLE with clear surgical margins or MMS (Figure 1B–D). Adjuvant radiotherapy and targeted therapy may be used to reduce the incidence of both local recurrence and metastasis [101].

### 9.3. Treatment of Unresectable/Metastatic DFSP

Unresectable DFSPs include advanced stage tumors, recurrent tumors without any possibility for further resection due to the size and/or location, or tumors in which further resection is likely to cause severe functional or cosmetic defects as shown in Figure 2C,D, and multiple organ metastases. These tumors should be treated with adjuvant radiation and/or targeted therapy in consultation with a multidisciplinary specialized soft-tissue sarcoma tumor board. Metastatic DFSP may also be treated with single or multiple agent chemotherapy regimens that are used for sarcomas.

#### 9.3.1. Radiation Therapy

Multiple studies have shown that DFSP is a radio-responsive tumor and adjuvant radiation therapy is effective to control tumor growth and reduce the incidence of postoperative recurrence [102,103,104,105,106]. A retrospective analysis of 53 DFSP patients treated with surgery and either preoperative or postoperative radiation therapy in the MD Anderson Cancer Center showed disease-free survival rates of 98% and 93% at 5 and 10 years, respectively [105]. A total of 60 Gy for indeterminate or microscopic positive margins and up to 70 Gy for macroscopic positive margins or primary gross tumor should be given [86,94]. The radiation field should extend 3–5 cm beyond the surgical margins or primary tumor boundary whenever feasible. An individual dose can be given at 2 Gy daily, 5 times weekly [86,94].

#### 9.3.2. Targeted Therapy

Imatinib mesylate (IM) is a potent and specific protein tyrosine kinase inhibitor interfering with the phosphorylation and activation of the PDGF receptor β which is constitutively activated due to translocation and fusion between *PDGFB* and *COL1A1* genes as discussed in Pathogenesis section. Recent in vitro and in vivo studies demonstrated that IM had growth-inhibitory effects on DFSP [60,61]. The effectiveness was evidenced by decreased tumor cellularity and formation of strong hyalinic fibrosis in tissues responded to IM treatment [107]. Treatment with IM in FS-DFSP revealed significant upregulation of the cell-cycle inhibitor p21^Cip1^ and β-galactosidase (a marker of cellular senescence) but decreased Ki-67 [108]. These data suggest that IM therapy is involved in modulation of tumor cell senescent and proliferative activities. IM may also play a role in immune modulation in tumor tissues. IM treated FS-DFSP tumor tissues compared with untreated tissues exhibited increased infiltration of the CD4 and CD8 T-cells in accompany with increased upregulation of cytokines and chemokines including IL-6, transforming growth factor-β1, CXCL-1, IL-1β and IL-8 and CD163^+^CD14^+^ myeloid cells transforming to express CD209 [108]. CD163^+^ macrophages are known to downregulate immune response whereas CD209^+^ myeloid cells promote T-cell-mediated antitumor responses. These data suggest that IM may exert direct antitumor effects via targeting the PDGFR pathway and indirect antitumor effects via T-cell mediated immune-modulation.

The efficacy of IM was observed in localized and metastatic DFSP with t(17; 22), but not in FS-DFSP lacking t(17; 22) [39,109,110,111]. A systemic review of IM treatment of 152 older patients (mean age: 49.3 years) with locally advanced (mean size: 9.9 cm in diameter) or metastatic DFSPs revealed 5.2% of patients with complete response (CR), 55.2% with partial response (PR), 27.6% in stable condition (SC) and 9.2% with progression. There were no differences in response rate using 400-mg or 800-mg daily doses for those with complete or partial responses [112]. A multicenter phase II trial of neoadjuvant IM therapy in advanced primary or locally recurrent DFSP resulted in 7.1% patients with CR, 50.0% with PR, 35.7% with SC and 7.1% with progression [107].

Further studies showed that IM used as a preoperative adjuvant therapy in DFSP lead to median tumor volume shrinkages from 20% to 31.5% [39,107,113,114], which transformed very large, unresectable tumors into resectable ones. Wang et al. reported four of 10 patients with primarily unresectable DFSP received complete surgical resection following IM treatment [38]. These studies provide new treatment options for patients with unresectable, recurrent, advanced and metastatic DFSP.

It should be noted that about 10% of DFSPs do not respond to IM treatment [38,107,112,115]. Some patients initially responding to IM treatment develop secondary resistance to IM rapidly [115,116]. Therefore, surgical excision for advanced primary tumor following IM therapy should be performed expeditiously during the “shrinkage window”.

The mechanism of resistance to IM is not clear yet. A low PDGFR phosphorylation level observed in IM resistant tumor tissues may not respond to IM [107,115]. Sunitinib, with its binding capacity of 10 times greater than that of IM, was demonstrated to be effective in IM resistant DFSP patients [107,115]. However, this effect may not be solely due to inhibiting PDFGR since Sunitinib is a multikinase inhibitor targeting not only PDGFR, but also vascular endothelial growth factor receptors 1–3, KIT, colony stimulating factor-1 receptor and FMS-like tyrosine-kinase-3 (FLT3) [117]. Other gene mutations or signal pathways may play a role in IM resistance. Whole-genomic sequencing in a patient with resistance to IM identified 8 nonsynonymous somatic gene mutations, including *ACAP2*, *CARD10*, *KIAA0556*, *PAAQR7*, *PPP1R39*, *SAFB2*, *STARD9* and *ZFYVE9* [118]. Single nucleotide polymorphism array and sequencing analysis of DFSP105 (an imatinib-resistant human cell line established from a FS-DFSP) showed a localized 9p21 homozygous deletion, encompassing *CDKN2A* and *CDKN2B*, which encode p14^ARF^, p15^INK4b^ and p16^INK4a^ [119]. p16 inhibits CDK4/6 activity. Loss of p16 expression leads to unrestricted cell cycle progression. Selection of new clonal cells after treatment with either immunotherapy or radiotherapy may also take a part in IM resistant treatment [120].

In the cases of IM resistance, other multikinase inhibitors, including sunitinib [107], sorafenib [121] and pazopanib [122], can be considered since treatment with these inhibitors showed effectiveness in IM resistant DFSP patients. In vitro and in vivo studies demonstrated the effectiveness of CDK4/6 inhibitors PD-0332991 and LEE011 in inhibiting DFSP105 proliferation, suggesting that CDK4/6 inhibitors can be potential drugs in p16 negative FS-DFSP [119]. Loss of p16 expression was demonstrated in 2 of 12 classic DFSP and 2 of 6 FS-DFSP [119]. Programmed cell death 1 ligand (PD-L1) expression was detected in metastatic FS-DFSP, but not in the primary tumor, suggesting a role of PD-L1 in the metastasis of FS-DFSP [123]. Since PD-L1 expression in the tumor cells is involved in immune escape from T cell attack [124], PD-L1 signal pathway may be a potential target for metastatic FS-DFSP.

## 10. Prognosis and Surveillance

The prognoses of the patients with DFSP, after surgical resection with negative and sometimes even positive microscopic margins, are generally good. The five- and ten-year recurrence-free survival rates of DFSP are 86% and 76% [44], respectively. Increased age, high mitotic index, positive margins and increased cellularity are predictors of poor clinical outcome [43]. Local recurrence is a major concern after surgical excision. The frequencies of local recurrences ranged from 20% to 50% [41,44], especially with positive margins. The median time from excision to local recurrence was reported from 32 to 38 months [43,44]. Therefore, mandatory long-term surveillance, at an interval of 6 to 12 months, is recommended [43]. Several studies suggest patients should be reevaluated every 6 months for the first five years and then yearly thereafter [86,99]. A thorough history and clinical examination of the primary sites and draining lymph nodes should be performed at each visit. Further imaging examinations should be considered based on patient’s tumor’s clinical manifestations (size, site, location, rate of growth), surgical procedures and histopathology (presence of high-risk features and margin status). Biopsy should be done on suspected local or distant recurrences or lymph node metastasis.

## 11. Conclusions

DFSP is a low- to intermediate-grade malignancy frequently occurring in the young to middle-aged population. It is histologically characterized by bland spindle cells in a storiform pattern with multiple variants. This needs to be differentiated from other benign and malignant lesions. A vast majority harbor t(17; 22) (q22; q13) resulting in the formation of *COL1A1-PDGFB* fusion gene transcript, which holds not only diagnostic value, but also therapeutic significance. WLE with a safe margin should be selected for most DFSPs located on the trunk and extremities. MMS should be considered for relatively small DFSPs in cosmetically sensitive regions to achieve the best tissue preservation with more appealing cosmetic and functional recoveries and can also be used for medium to large DFSP of the trunk and extremities with high cure rates. Subtotal or total digital amputation should be considered for DFSPs located on the digits where complete surgical excision is impractical. Adjuvant therapies—including radiation and targeted therapy—should be chosen for the patients who are unsuitable for surgical excision. Periodic surveillance, at an interval of 6 months in the first five years and then yearly post-excision, is recommended to monitor potential recurrence and metastasis.

## Figures and Tables

**Figure 8:**
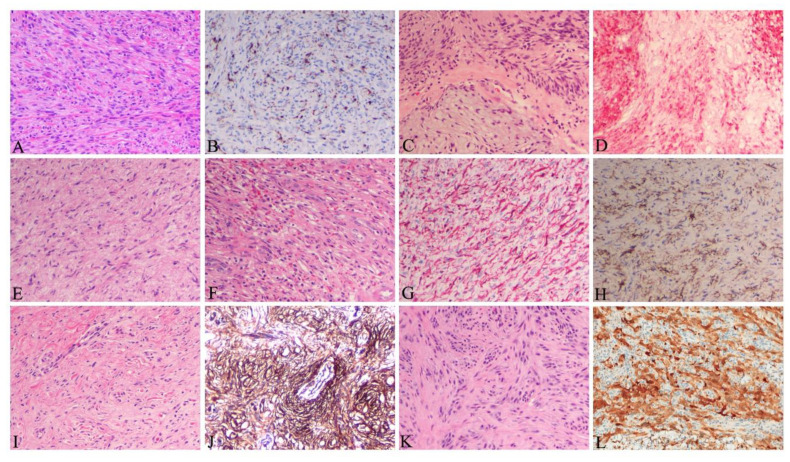
Histopathology of different tumors differentiated from dermatofibrosarcoma protuberans. (**A**) Dermatofibroma: Interlacing fascicles of spindle shaped fibroblasts and histiocytic cells mixed with collagens (HE, 100 × 1); (**B**) factor XIIIa expression in dermatofibroma (same tissue as A, DAB, 100 × 1); (**C**) schwannoma: wavy hyperchromatic spindle cells arranged in palisades (Antoni A, upper part) and myxoid hypocellular components (Antoni B, lower part) (HE, 100 × 1); (**D**) S-100 expression in schwannoma (same tissue as C, alkaline phosphatase red, 100 × 1); **(E)** neurofibroma: bland serpentine spindle shaped cells and shredded carrot collagens (HE, 100 × 1); **(F)** neurofibroma: mast cells and lymphocytes interspersed among the spindle cells and tumor stroma (same tissue as E, HE, 100 × 1); (**G**) S-100 expression in neurofibroma (same tissue as E, alkaline phosphatase red, 100 × 1); (**H**) CD34 expression in neurofibroma (same tissue as E, DAB, 100 × 1); (**I**) spindle cell lipoma: bland spindle cells without matured lipocytes embedded in ropey/refractile collagen bundles (HE, 100 × 1); (**J**) CD34 expression in spindle cell lipoma (same tissue as I, DAB, 100 × 1); (**K**) melanoma: spindle shaped cells with light scattered pigments (HE, 100 × 1); (**L**) S-100 expression in spindle cell melanoma (DAB, 200 × 1).

**Table 1:** Histopathologic features of different dermatofibrosarcoma protuberans variants.

Variants.	Histopathologic Features
Myxoid	Elongated, infiltrative spindle cells with myxoid changes in stroma (Figure 7E); CD34^+^, alpha smooth muscle actin^-^_,_ desmin^-^
Pigmented (Bednář tumor)	Spindle cells admixed with scattered, single or a small cluster of dendritic melanin-containing cells [69]; CD34^+^; S-100^+^ and HBM45^+^ in pigmented cells
Giant cell (Rare)	Spindle cells admixed with pleomorphic or multinucleated giant cells (Figure 7F) [70,71]; CD34^+^
giant cell fibroblastoma (GCF) (commonly seen in the pediatric population)	Parallel fascicles of wavy uniform spindled cells with wiry collagen, dense sclerosis and pseudovascular spaces with scattered and rimming pleomorphic giant cells [71]. GCF shares the same genetic abnormality as DFSP and the recurrent cases of GCF show histological features of DFSP [72,73,74,75,76]
Granular cell (Rare)	Spindle cells admixed with a proportion of cells with eccentric round nuclei, prominent nucleoli and abundant lysosomal granules [77]; CD34^+^, natural killer cell inhibitory factor 1C3^+^
Sclerotic (Rare)	Spindle cells embedded in more than half of hypocellular collagenous components [78] (Figure 7G), CD34^+^
Fibrosarcomatous (13.5%) [66]	Increased spindle cells with atypia, increased mitotic figures; fascicular or herringbone rather than storiform pattern; necrosis occasionally observed (Figure 7H) [66]; reduced or even lost CD34 expression (Figure 7I) [13]

**Table 2:** Differential diagnosis of dermatofibrosarcoma protuberans.

Tumor	Clinical Feature	Histology	Immunostain
Dermatofibroma [80]	Elevated, pedunculated or dome shaped. More frequent in extremities, young (20–49 years) and females predominant	More pleomorphic with both small spindle-shaped fibroblastic cells and larger histiocytes admixed with chronic inflammatory cells in dermis (Figure 8A). Hyperkeratosis, acanthosis and pigmentation in epidermis.	XIIIa^+^ (Figure 8B) CD34^-^
Schwannoma [81]	Round, ovoid, well-circumscribed, solid mass, most common on the limbs, between 20–50 years	Well circumscribed with fibrous capsule, biphasic growth patterns with Antoni A (highly ordered wavy hyperchromatic spindle cells arranged in palisades) and with Antoni B (myxoid hypocellular components) (Figure 8C)	S100^+^(Figure 8D)
Cutaneous neurofibroma [82]	Skin colored, painless, slowly growing, solitary, soft, rubbery nodule. Frequently occurred in younger patients (20 to 40 years)	Mixed multiple cell types including Schwann cells, perineurial-like cells, fibroblastic cells, entrapped axons in interspersed with shredded carrot collagen, mast cells and lymphocytes (Figure 8E,F)	S-100^+^ (Figure 8G), Sox10^+^, CD34^+^ (Figure 8H), Collagen IV^+^, αSMA^-^, XIIIa^-^
Solitary fibrous tumor [83]	Usually occurred in older adults, slow-growing and painless mass with low rate of infiltration and metastasis	Relatively bland and uniform spindle cells within long, thin and parallel bands of collagen in “patternless” arrangement	CD34^+^, CD99^+^, STAT6^+^ [83], Vimentin^+^, Desmin^-^, S100^-^
Intradermal spindle cell lipoma [84]	Slowly growing, skin colored, raised, polypoid lesion with well-defined margin in seniors.	Bland spindle cells admixed with more or less or no matured lipocytes associated with delicate ropey/refractile collagen bundles (Figure 8I)	CD34^+^(Figure 8J), Rb^-^, S-100^-^, αSMA^-^
Spindle cell/ desmoplastic melanoma [85]	Pigmented or non-pigmented nodule on sun exposed skin in older adults	Significant atypia, pleomorphism, nuclear hyperchromasia, lack of storiform arrangement, pigmentations in spindle cells, derived from dysplastic melanocytic cells (Figure 8K)	S-100^+^(Figure 8L), typically Melan-A^-^and HMB-45^-^, sometimes, CD34^+^

**Table 3:** Staging system of dermatofibrosarcoma protuberans.

Stage	Criteria
Stage I	Non-protuberant lesions including atrophic or sclerotic plaque, macula or small nodules
Stage II	Protuberant primary tumor
Stage IIA	Superficial tumor: without invasion of the underlying fascia
Stage IIB	Deep tumor: either superficial to the fascia with infiltrating the fascia or occurred beneath the superficial fascia
Stage III	Lymph node metastasis
Stage IV	Distant metastasis to other organs

**Table 4:** Comparison between wide local excision (WLE) and Mohs micrographic surgery (MMS).

Comparable Parameter	WLE	MMS
Surgical procedures [87,88]	A three-dimensional excision including normal skin, subcutaneous tissue and the underlying investing fascia within a 2–4 cm margins from the gross tumor boundary	A stepwise procedure of tumor removal with mapping, histopathologic examination of 100% of the margins with tangential frozen sections by the Mohs surgeon and further deeper and/or wider re-excision of another layer of surrounding tissues if residual tumor cells are visualized. This procedure is repeated until all tumor margins are free of tumor cells. Usually performed as an outpatient with local anesthesia (Figure 9)
Advantages [87]	Relative simpler procedure Immediate wound repair following tumor removal Cost effective for patients and medical resources (but additional cost is incurred if positive margins need to be addressed)	Precise and complete evaluation of 100% of the surgical margins during excision; wound repair done when clear margins are obtained Lower rates of local recurrence compared with surgical excision
Drawbacks [87]	Unable to evaluate surgical margins during surgical operation Higher rate of local recurrences compared to Mohs surgery	Needs specialized training for Mohs surgeons and coordination with in office histotechnologists Delayed closure (usually same day) to allow for pathologic evaluation Time consuming and labor intensive May have higher cost for patients and medical resources
Applications [89]	Best for primary DFSPs on the trunk or extremities with a 2–4 cm margin from tumor boundary to completely excise tumor with acceptable cosmesis and function in a single operation	Ideal for DFSPs in cosmetically and functionally sensitive regions including face, scalp, neck, genitalia and digits to preserve tissue for optimal cosmetic reconstruction and functional recoveries and may be utilized in trunk and extremity DFSP
